# Long noncoding RNA FAM225A promotes the malignant progression of gastric cancer through the miR-326/PADI2 axis

**DOI:** 10.1038/s41420-021-00809-1

**Published:** 2022-01-11

**Authors:** Xiang Ma, Gang Wang, Hao Fan, Zengliang Li, Wangwang Chen, Jian Xiao, Peidong Ni, Kanghui Liu, Kuan Shen, Yuanhang Wang, Zekuan Xu, Li Yang

**Affiliations:** 1 Department of General Surgery, The First Affiliated Hospital of Nanjing Medical University, Nanjing, Jiangsu China; 2 Department of General Surgery, Liyang People’s Hospital, Liyang Branch Hospital of Jiangsu Province Hospital, Liyang, Jiangsu China

**Keywords:** Gastric cancer, Gastric cancer

## Abstract

Gastric cancer (GC) is a global health problem and further studies of its molecular mechanisms are needed to identify effective therapeutic targets. Although some long noncoding RNAs (lncRNAs) have been found to be involved in the progression of GC, the molecular mechanisms of many GC-related lncRNAs remain unclear. In this study, a series of in vivo and in vitro assays were performed to study the relationship between FAM225A and GC, which showed that FAM225A levels were correlated with poor prognosis in GC. Higher FAM225A expression tended to be correlated with a more profound lymphatic metastasis rate, larger tumor size, and more advanced tumor stage. FAM225A also promoted gastric cell proliferation, invasion, and migration. Further mechanistic investigation showed that FAM225A acted as a miR-326 sponge to upregulate its direct target PADI2 in GC. Overall, our findings indicated that FAM225A promoted GC development and progression via a competitive endogenous RNA network of FAM225A/miR-326/PADI2 in GC, providing insight into possible therapeutic targets and prognosis of GC.

## Introduction

Gastric cancer (GC) is mainly concentrated in Asia, including China, Japan, and the Republic of Korea. Nearly 50% of patients with GC in China are at a locally advanced stage, which seriously affects national health [1, 2]. The primary treatment of GC has been surgical resection; however, after decades of clinical observation, it has been found that although surgical resection can significantly improve survival and quality of life of early-stage GC patients, the prognosis of patients with progressive GC are still not ideal, and there is still a high local recurrence rate after surgery [3, 4]. At present, we know little about the cellular and molecular mechanisms of gastric carcinogenesis. Therefore, studying the pathogenesis of GC and identifying new biomarkers with therapeutic potential is important [5].

Long noncoding RNAs (lncRNAs) are more than 200 nucleotides in length, are functionally cataloged as noncoding transcripts, and are subject to wide transcription in the genome [6, 7]. In recent years, mounting studies have found that abnormally expressed lncRNAs play a significant role in the tumorigenesis and development of a variety of tumors, including GC [8–10]. However, unlike other noncoding RNAs (ncRNAs), such as small interfering RNAs (siRNAs) and microRNAs (miRNAs), the regulatory mechanisms of lncRNAs appear to be more complex. lncRNAs function in the cell by epigenetic, transcriptional, or posttranscriptional mechanisms [11–13]. Through these mechanisms, lncRNAs act as oncogenes or tumor suppressor genes to regulate tumor cell proliferation, metastasis, and drug resistance [14]. The complex interactions between mRNA and ncRNAs, such as lncRNAs, have been described as competitive endogenous RNA (ceRNA) networks [15, 16]. The ceRNA hypothesis indicates lncRNAs can competitively bind miRNAs to affect the expression level of miRNA target genes by posttranscriptional regulation [17–19]. Although multiple lncRNAs have been identified as regulators in tumor pathogenesis, the function of lncRNAs as miRNA sponges has not been clearly elucidated in GC progression. The lncRNA-mediated ceRNA molecular patterns have revealed a novel approach for understanding the specific mechanism of GC development and for identifying potential diagnostic or therapeutic targets.

In the present study, the GC-associated lncRNA FAM225A was identified and shown to be highly expressed with poor prognosis in GC. Notably, the biological functions played by FAM225A in GC have not been investigated to date. Subsequently, we discovered that higher FAM225A expression was positively associated with a more profound lymphatic metastasis rate, larger tumor size, and more advanced tumor stage. Functionally, a series of in vitro and in vivo assays showed that FAM225A promoted gastric cell proliferation, invasion, and migration. Mechanistically, we found that FAM225A acted as a miR-326 sponge to upregulate its direct target PADI2 in GC. Overall, our findings revealed a novel ceRNA network of FAM225A/miR-326/PADI2 in GC, providing insight into possible therapeutic targets and prognosis prediction of GC.

## Results

### FAM225A is upregulated in GC and predicts a poor prognosis

**Fig. 1:**
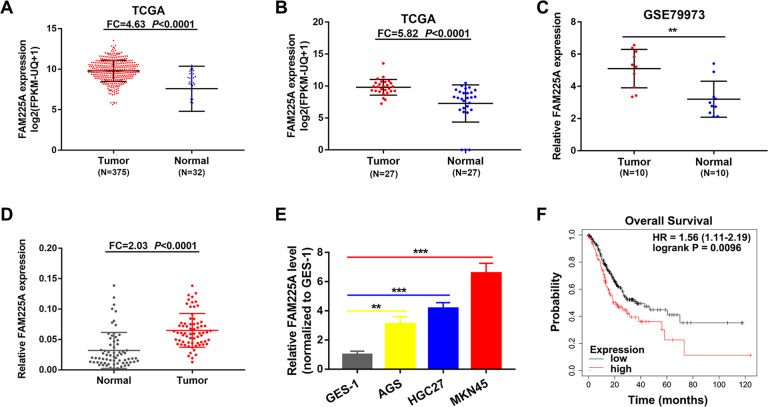
FAM225A is upregulated in GC and predicts a poor prognosis. **A**, **B** Analyzing the expression of FAM225A in unpaired and paired GC tissues by using TCGA database. **C** Relative mRNA expression of FAM225A in GC tissues according to GEO database. **D**, **E** The expression of FAM225A was detected in our own 68 paired GC tissues, GC cells and GES-1. **F** Survival analysis of the association between FAM225A level and the overall survival (OS) rate of GC patients. *p* values were obtained by Student’s *t* test (**A**–**C**, **E**) or Wilcoxon test (**D**). FC fold change, **p < 0.01, ***p < 0.001.

FAM225A was identified by analyzing the human microarray dataset GSE79973 from the Gene Expression Omnibus (GEO) database and RNA sequencing (RNA-seq) data from The Cancer Genome Atlas (TCGA). These data suggested FAM225A was highly expressed in unpaired and paired GC tissues (Fig. 1A–C). Subsequently, the expression of FAM225A was detected in our own 68 paired GC tissues, and GC and GES-1 cells. We discovered that FAM225A was upregulated compared with normal tissues and GES-1 cells (Fig. 1D, E). The correlation between FAM225A levels and patient clinicopathological characteristics suggested that higher FAM225A expression tended to correlate with more profound lymphatic metastasis rate, larger tumor size, and more advanced tumor stages (Table S1). Furthermore, Kaplan–Meier analysis exposed a negative association between FAM225A level and the overall survival rate of GC patients (Fig. 1F). These findings revealed that FAM225A was highly expressed and correlated with a poor prognosis in GC.

### FAM225A promotes GC cell proliferation and induces cell apoptosis

**Fig. 2:**
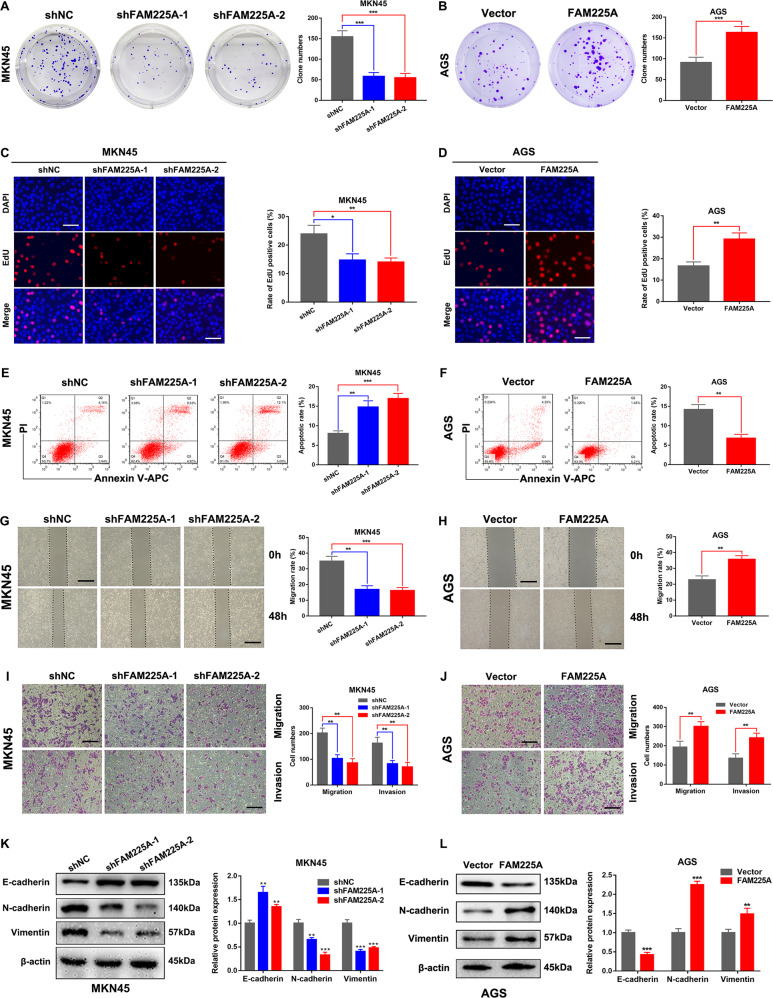
FAM225A promotes GC cell proliferation, migration, and invasion and induces cell apoptosis. **A**–**D** Measuring MKN45 or AGS cell proliferation ability when knockdown or overexpression of FAM225A through colony formation assays and the Edu assays (scale bar: 100 μm for Edu assay). **E**, **F** Flow cytometric analysis of MKN45 or AGS cells by downregulating or upregulating FAM225A expression to detect the cell apoptosis. **G**–**J** Wound healing assay and Transwell assay revealed that silencing FAM225A could dramatically attenuate MKN45 cells migration ability, while overexpression of FAM225A has the opposite effects in AGS cells (scale bar: 200 μm for transwell assay, 100 μm for wound healing assay). **K**, **L** Western blot analysis of the EMT-related molecules in GC cells when knockdown or overexpression of FAM225A. *p* values were obtained by Student’s *t* test. Data are presented as means ± SD. *p < 0.05, **p < 0.01, ***p < 0.001.

To investigate the biological function of FAM225A in GC, we transfected MKN45 and AGS cells to downregulate or upregulate FAM225A expression. Transfection efficiency was verified by qRT-PCR (Fig. S1A, B). A colony formation assay indicated that knockdown of FAM225A inhibited MKN45 cell proliferation and overexpression of FAM225A promoted AGS cell proliferation (Fig. 2A, B). The results of the EdU assay were consistent with those of the colony formation assay (Fig. 2C, D). Flow cytometric analysis of GC cells revealed that MKN45 cell apoptosis increased following upregulation of FAM225A expression and AGS cell apoptosis decreased following downregulation of FAM225A expression (Fig. 2E, F). These results suggested that FAM225A could promote cell proliferation and induce apoptosis in GC.

### FAM225A promotes migration and invasion of GC cells

Subsequently, we employed a wound healing assay, Transwell assay, and western blotting to investigate whether FAM225A plays a role in GC cell migration and invasion. The wound healing assay showed that silencing FAM225A dramatically attenuated MKN45 cell migration ability compared with the negative control, while overexpression of FAM225A had the opposite effect in AGS cells (Fig. 2G, H). The results of the Transwell migration and invasion assay were consistent with those of the wound healing assay (Fig. 2I, J). The epithelial-mesenchymal transition (EMT) is activated during tumor cell metastasis and is considered the main pathological event of tumorigenesis [20, 21]. Western blot analysis of EMT-related proteins showed that knockdown of FAM225A in MKN45 cells was accompanied by decreased expression of vimentin and N-cadherin, and increased expression of E-cadherin. Opposite results were observed in AGS cells (Fig. 2K, L). Collectively, these data revealed that FAM225A promoted migration and invasion of GC cells.

### Knockdown of FAM225A inhibits GC tumor growth and metastasis in vivo

**Fig. 3:**
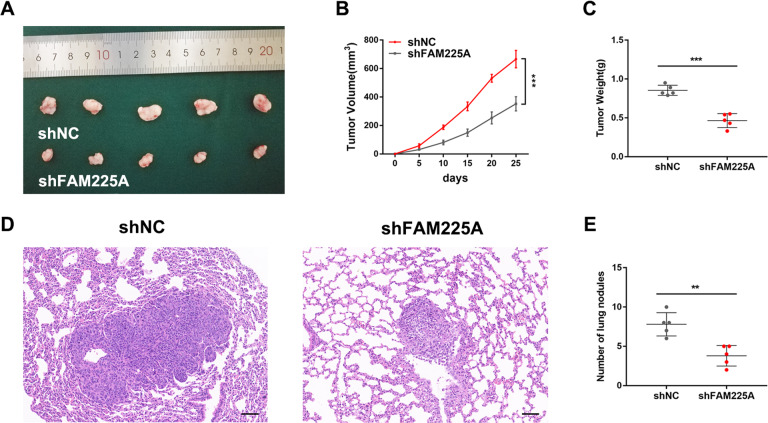
Knockdown of FAM225A inhibits GC tumor growth and metastasis in vivo. **A** Tumor tissues harvested from shFAM225A and shNC groups of female nude mice. **B** Tumor volumes in both groups were measured every 5 days. **C** Tumor weight in different groups. **D**, **E** HE staining of specimen to show lung metastasis in shFAM225A and shNC groups (scale bar: 50 μm). *p* values were obtained by Student’s *t* test (**C**, **E**) or ANOVA (**B**). Data are presented as means ± SD. **p < 0.01, ***p < 0.001.

To determine whether FAM225A could affect tumor growth in vivo, AGS cells stably transfected with shRNA targeting FAM225A or their negative control groups were inoculated subcutaneously into female nude mice. Tumor size in shFAM225A groups were relatively smaller and the weights of tumor explants were also smaller compared with control groups (Fig. 3A–C). Meanwhile, the tumor metastasis model assay in vivo showed that lung metastasis in the FAM225A knockdown group was alleviated (Fig. 3D, E). These results indicated that FAM225A inhibited GC tumor growth and metastasis in vivo.

### FAM225A functions as a ceRNA and competitively binds miR-326 in GC

**Fig. 4:**
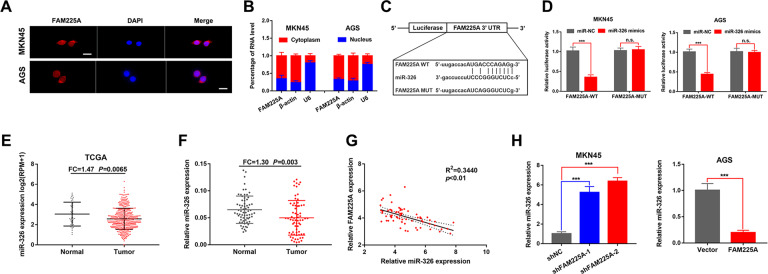
FAM225A functions as a ceRNA and competitively absorbs miR-326 in GC. **A**, **B** FISH and qRT-PCR analysis of the subcellular localization of FAM225A in gastric cancer cells (scale bar: 50 μm for FISH assay). **C**, **D** A dual-luciferase reporter assay suggested that the relative luciferase activity of the wild-type FAM225A was reduced by miR-326. **E**, **F** Relative expression of miR-326 in GC tissues according to TCGA database and our own 68 GC tissues. **G** The correlation between the expression of FAM225A and miR-326 was analyzed in 68 GC tissues. **H** Detecting miR-326 expression when knockdown or overexpression of FAM225A in MKN45 or AGS cells by using qRT-PCR. *p* values were obtained by Student’s *t* test. Data are presented as means ± SD. FC fold change, ***p < 0.001.

To explore the underlying molecular mechanism of FAM225A in GC, we determined the subcellular localization of FAM225A. FISH and subcellular fractionation analysis of GC cells revealed that FAM225A was mainly localized in the cytoplasm, indicating FAM225A might function as a ceRNA of miRNAs and post-transcriptionally regulate target gene expression (Fig. 4A, B). Subsequently, miR-326 was identified as a miRNA that may interact with FAM225A using the DIANA (http://carolina.imis.athena-innovation.gr/diana_tools/web/index.php) and lncRNASNP2 (http://bioinfo.life.hust.edu.cn/lncRNASNP#!/) databases [22, 23]. A dual-luciferase reporter assay further suggested that the relative luciferase activity of wild-type FAM225A was reduced in the miR-326 mimics group (Fig. 4C, D). We found that miR-326 was downregulated via analysis of the TCGA database and our own 68 GC tissues, which suggested that miR-326 might play a role as a tumor suppressor gene in GC (Fig. 4E, F). Besides, miR-326 expression was negatively association with FAM225A levels in GC tissues (Fig. 4G). miR-326 expression was also markedly enhanced in the FAM225A knockdown group and significantly impaired in the FAM225A-overexpression group (Fig. 4H). Overall, FAM225A suppressed miR-326 expression in GC cells by acting as a molecular sponge.

### FAM225A modulates GC cell proliferation, migration, and invasion by negatively regulating miR-326

**Fig. 5:**
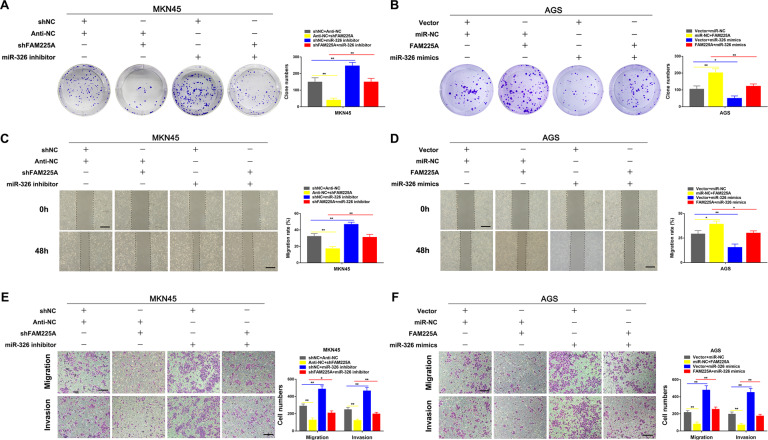
The functional effects of FAM225A can be regulated by miR-326 in GC. **A**, **B** Colony formation assays was used to detect the cell proliferation ability after transfecting MAN45 cells with negative control, shFAM225A, miR-326 inhibitor or shFAM225A + miR-326 inhibitor, and transfecting AGS cells with negative control, FAM225A, miR-326 mimics or FAM225A + miR-326 mimics. **C**–**F** The wound healing assays and transwell assays were used to detect the cell migration and invasion ability after transfecting GC cells (scale bar: 100 μm for wound healing assay, 200 μm for transwell assay). *p* values were obtained by Student’s *t* test. Data are presented as means ± SD. *p < 0.05, **p < 0.01.

To study whether FAM225A functioned via miR-326 in GC, we first intervened with FAM225A expression and downregulated or upregulated miR-326 using miR-326 mimics or inhibitors in GC cells. Using colony formation, wound healing and transwell assays, we discovered that FAM225A knockdown-mediated inhibition of MKN45 cell proliferation, migration and invasion could be partially rescued by silencing miR-326 (Fig. 5A, C, E), and that miR-326 mimics could partially rescue FAM225A-overexpression-mediated promotion of AGS cell proliferation, migration and invasion (Fig. 5B, D, F). These results indicated that FAM225A modulated GC cell proliferation, migration and invasion by negatively regulating miR-326.

### FAM225A modulates PADI2 expression by competitively binding miR-326

**Fig. 6:**
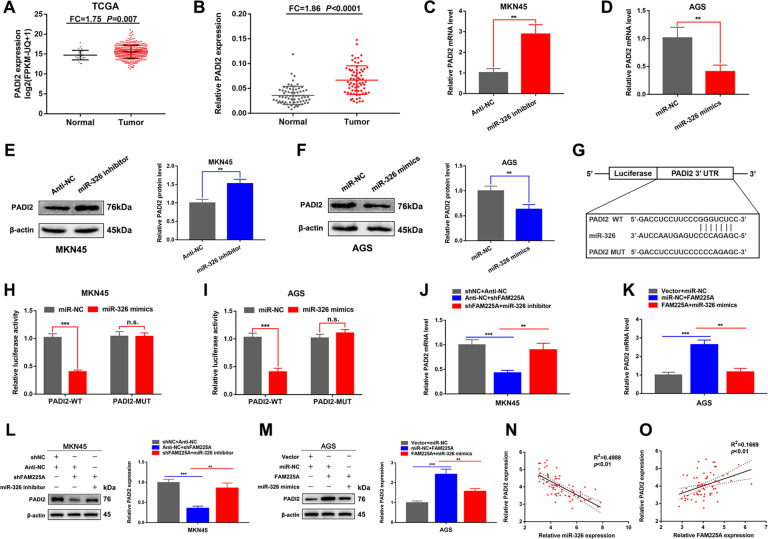
FAM225A modulates PADI2 expression by competitively binding miR-326. **A**, **B** Relative expression of PADI2 in GC tissues according to TCGA database and our own 68 GC tissues. **C**–**F** Detecting PADI2 mRNA and protein expression when knockdown or overexpression of miR-326 in MKN45 or AGS cells by using qRT-PCR and western blot. **G** Predicting binding sites between PADI2 and miR-326. **H**, **I** The relative luciferase activity of the wild-type PADI2 was reduced by upregulating miR-326. **J**–**M** Detecting PADI2 mRNA and protein expression when MKN45 cells transfected with siNC + Anti-NC, Anti-NC + siFAM225A, siFAM225A + miR-326 inhibitor, or when AGS cells transfected with Vector + miR-NC, miR-NC + FAM225A, FAM225A + miR-326 mimics by using qRT-PCR and western blot. **N**, **O** The correlation between the expression of PADI2 and the expression of miR-326 or FAM225A was analyzed in 68 GC tissues. *p* values were obtained by Student’s *t* test. Data are presented as means ± SD. FC fold change, **p < 0.01, ***p < 0.001.

To explore the targets of miR-326 and the ceRNA lncRNA/miR-RNA/mRNA network in GC, we predicted putative targets of miR-326 using the miRDB (http://mirdb.org/mirdb/index.html) and TargetScan (http://www.targetscan.org/vert_71/) databases [24, 25], which suggested that PADI2 maybe the direct target of miR-32. Analysis of the TCGA database and our 68 GC tissues revealed that PADI2 was upregulated, suggesting PADI2 may also function as an oncogene in GC (Fig. 6A, B). Further detection of mRNA and protein levels of PADI2 showed that PADI2 expression was decreased following co-transfection with miR-326 mimics and increased following co-transfection with miR-326 inhibitor in GC cells (Fig. 6C–F). A dual-luciferase reporter assay also suggested that miR-326 mimics could reduce the relative luciferase activity of wild-type PADI2 (Fig. 6G–I). For the rescue experiment, the decrease in PADI2 mRNA or protein expression induced by knockdown of FAM225A was partially counteracted by co-transfection with the miR-326 inhibitor in MKN45 cells. AGS cells showed opposite results following transfection of miR-326 mimics with overexpression of FAM225A (Fig. 6J–M). Moreover, a negative and positive correlation was observed between PADI2 and miR-326 and FAM225A, respectively (Fig. 6N, O). Together, our results suggested FAM225A modulated PADI2 expression by competitively binding miR-326.

### Regulation of PADI2 in GC cells is mediated by miR-326

To investigate the function of PADI2 and miR-326 in GC and their relationship, we downregulated or upregulated PADI2 or miR-326 in MKN45 or AGS cells, respectively. Colony formation, wound healing and transwell assays showed that miR-326 inhibitor could partially rescue knockdown of PADI2-mediated inhibition of MKN45 cell proliferation, migration and invasion. AGS cells with overexpression of PADI2 and miR-326 showed oppositive results (Fig. S2A–F). These experiments revealed that the carcinogenic effect of PADI2 in GC was partially mediated by miR-326.

### Effects of FAM225A on GC cell proliferation, migration, and invasion could be reversed by PADI2

**Fig. 7:**
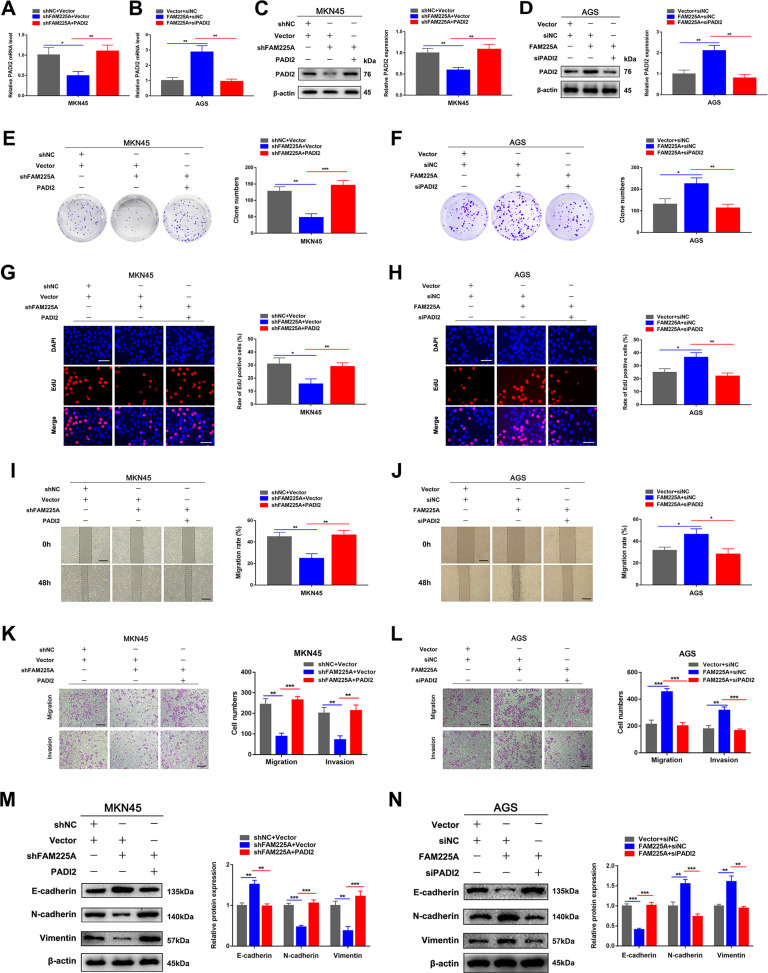
The effect of FAM225A on GC cell proliferation, migration, and invasion could be reversed by PADI2. **A**–**D** Detecting PADI2 mRNA and protein expression when MKN45 cells transfected with siNC + Vector, siFAM225A + Vector, siFAM225A + PADI2, or when AGS cells transfected with Vector + siNC, FAM225A + siNC, FAM225A + siPADI2 by using qRT-PCR and western blot. **E**–**H** Colony formation assay and edu assay demonstrated that knockdown of FAM225A mediated inhibition of MKN45 cell proliferation were partially rescued by overexpression of PADI2, and silencing PADI2 could partially counteract overexpression of FAM225A mediated promotion of AGS cell proliferation (scale bar: 100 μm for Edu). **I**–**L** Detecting the cell migration and invasion ability after transfecting GC cells through wound healing assays and transwell assays (scale bar: 200 μm for transwell assay, 100 μm for wound healing assay). **M**, **N** Western blot analysis of the EMT-related molecules in different treatment groups. *p* values were obtained by Student’s *t* test. Data are presented as means ± SD. *p < 0.05, **p < 0.01, ***p < 0.001.

To further explore the biological interactions between FAM225A and PADI2 in GC, we transfected MKN45 cells with siFAM225A, PADI2-overexpression vector, or their negative control groups. We also transfected AGS cells with FAM225A-overexpression vector, siPADI2, or the corresponding empty vector. The qRT-PCR and western blotting were performed to verify the transfection efficiency (Fig. 7A–D). Colony formation, EdU, wound healing, and Transwell assays demonstrated that overexpression of PADI2 could partially counteract FAM225A knockdown-mediated inhibition of MKN45 cell proliferation, migration, and invasion, and silencing of PADI2 could partially counteract FAM225A-overexpression-mediated promotion of AGS cell proliferation, migration, and invasion (Fig. 7E–L). Western blot analysis of EMT-related proteins indicated that downregulation or upregulation of FAM225A or PADI2 in GC cells could alter the protein expression of vimentin, N-cadherin, and E-cadherin, which was consistent with the Transwell assay results (Fig. 7M, N). Thus, altering the expression of PADI2 could reverse the effect of FAM225A on GC cell proliferation, migration, and invasion.

## Discussion

GC remains a global health problem, and further studies on the molecular mechanisms of GC are needed to identify novel therapeutic targets. Most human genomes are transcribed into ncRNAs, whereas protein coding genes account for a small proportion; this has been confirmed by the Encyclopedia of DNA Elements (ENCODE) Project Consortium [26]. The potential value of ncRNAs, especially miRNAs and lncRNAs, in improving the diagnosis and treatment of GC has been confirmed by an increasing number of studies [27, 28]. Although some lncRNAs have been found to be involved in the progression of GC, the molecular mechanisms of many lncRNAs related to GC are still unknown. The present study showed that the lncRNA FAM225A was overexpressed in GC, which may promote GC progression by regulating the miR-326/PADI2 axis.

TCGA project and GEO database provide large-scale molecular distortion data of DNA, RNA, protein, and epigenetic levels in various types of tumors that aid in understanding potential carcinogenic molecular mechanisms; this data is widely used in studies of various tumors including GC [29–32]. In the present study, we first identified the GC-associated lncRNA FAM225A by analyzing data from TCGA and the GEO database, which suggested that FAM225A was overexpressed in GC tissues. We continued to confirm the high expression of FAM225A in our own tissues and cells. Clinicopathological analysis indicated that increased expression of FAM225A was associated with a higher lymph node metastasis rate, larger tumor size, and more advanced tumor stage, and the increased expression of FAM225A indicated poor prognosis in GC patients. Functionally, a series of in vitro and in vivo experiments revealed that FAM225A promoted gastric cell proliferation, invasion, and migration.

It has been emphasized that the different biological functions of lncRNAs depend to a large extent on their subcellular localizations [33]. Cytoplasmic lncRNAs can act as decoys for miRNAs to regulate the stability or translation of mRNA, affecting signaling pathways [34], suggesting lncRNAs are involved in posttranscriptional regulation [13]. The complex interactions between mRNA and lncRNAs have been described as ceRNA networks [15, 16]. For example, LINC00857 contributes to diffuse large B-cell lymphoma proliferation and lymphomagenesis by sponging miR-370-3p [35], while lncRNA MIR17HG promotes colorectal cancer liver metastasis by sponging miR-138-5p [36]. The lncRNA NKX2-1-AS1 promotes tumor progression and angiogenesis by sponging miR-145-5p in GC [37], while LINC01234 promotes GC cell proliferation and inhibits cell apoptosis by sponging miR-204-5p [32]. Besides, FAM225A was found to facilitate colorectal cancer progression by sponging miR-613 [38]. In the present study, we speculated that FAM225A might function as a ceRNA of miRNAs and post-transcriptionally regulate expression of target genes based on its cytoplasmic localization. FAM225A was proven to bind miR-326 by bioinformatics analyses (DIANA (http://carolina.imis.athena-innovation.gr/diana_tools/web/index.php) and lncRNASNP2 (http://bioinfo.life.hust.edu.cn/lncRNASNP#!/)) and luciferase reporter assays. miR-326 has been confirmed as a tumor suppressor gene in a variety of tumors including GC [39–41]. We also observed that miR-326 was downregulated in GC, and miR-326 expression was negatively associated with FAM225A levels. With gain or loss function assays, we revealed that FAM225A modulated GC cell proliferation and migration by negatively regulating miR-326. In consequence, it raises the possibility that FAM225A could be a sponge for other miRNAs and may therefore have other effects in different tumors.

According to the ceRNA hypothesis that lncRNAs competitively bind miRNAs to affect the expression of miRNA target genes at the posttranscriptional level [17–19], mRNA expression is upregulated as a result of the competitive binding of lncRNAs to miRNAs. The miRNAs play a role in cancer signaling pathways by inhibiting or degrading target genes, thus affecting cell fate and biological function [42]. In the present study, bioinformatics analyses (miRDB (http://mirdb.org/mirdb/index.html) and TargetScan (http://www.targetscan.org/vert_71/)) and luciferase reporter assays revealed that PADI2 was a direct target of miR-326. Although PADI2 has been reported to advance abnormal cell behavior in GC [43], how it works in the ceRNA network of FAM225A/miR-326/PADI2 has not been investigated. We found that PADI2 was upregulated, and a negative and positive correlation was observed between PADI2 and miR-326 and between PADI2 and FAM225A, respectively. Gain or loss function assays suggested that the carcinogenic effect of PADI2 was partially mediated by miR-326. Meanwhile, the effect of FAM225A on GC cell proliferation, migration, and invasion could be reversed by PADI2.

There were some limitations to this study that should be acknowledged. Due to the lack of follow-up information for our GC samples, we could not accurately confirm the relationship between FAM225A levels and survival prognosis in GC patients; we relied on the classic online database. Second, cell subcellular fractionation analysis showed that FAM225A was also partially expressed in the nucleus; its functional mechanism should also be explored. We will continue to explore these issues in future studies.

Overall, the lncRNA FAM225A acted as an oncogene to promote GC cell proliferation, invasion, and metastasis. Mechanistically, FAM225A promoted GC development and progression via a ceRNA network of FAM225A/miR-326/PADI2. Overall, our findings improved our understanding of the pathogenesis of GC and provided a theoretical basis for developing new and effective diagnostic treatment targets and/or reliable prognostic markers.

## Materials and methods

### Clinical specimens

All GC and matched normal control tissues in our study were obtained from GC patients who underwent gastrectomy in the General Surgery Department of the First Affiliated Hospital of Nanjing Medical University (NMU). All patients provided written informed consent. Ethical endorsement was attained from the clinical research Ethics Committee of the First Affiliated Hospital of NMU.

### Cell culture

All GC cells including AGS, HGC27, MKN45, and the human normal gastric epithelial cell line GES-1 were purchased from the Institute of Biochemistry and Cell Biology of the Chinese Academy of Sciences (Shanghai, China). RPMI-1640 medium (Gibco, USA) was used for MKN45 and GES-1 cells and F12K medium (Gibco, USA) was used for AGS and HGC27 cells; both were supplemented with 10% fetal bovine serum (FBS). All cells were cultured in a humidified incubator under 5% CO_2_ at 37 °C.

### Cell transfection

GenePharma (Shanghai, China) designed and synthesized the FAM225A/PADI2-overexpression plasmid, shFAM225A, siRNAs targeting FAM225A/PADI2, miR-326 mimics, miR-326 inhibitors, and their negative controls. Lipofectamine 3000 Reagent (Invitrogen, USA) was used to transfect above reagents according to the manufacturer’s recommendations. After transfection for 48 h, the transient transfection efficiency was assessed by qRT-PCR analysis. Stable cell lines were obtained by selection with puromycin.

### Quantitative real-time reverse transcription polymerase chain reaction (qRT-PCR) analysis

Complementary DNA was synthesized using a New Poly (A) Tailing Kit (Thermo Fisher Scientific, China) for miRNA and a Prime Script RT reagent Kit (Takara, China) for lncRNA/mRNA. Universal SYBR Green Master Mix (Roche, Shanghai, China) was used to perform the amplification with a 7500 Real-Time PCR System (Applied Biosystems, USA). Endogenous controls for mRNA and miRNA were β-actin and U6, respectively. Primer sequences were as follows: miR-326, forward: 5′-GCCGAGCCTCTGGGCCCTTC-3′, reverse: 5′-CAGTGCGTGTCGTGGAGT -3′; U6, forward: 5ʹ-CTCGCTTCGGCAGCACA-3ʹ, reverse: 5ʹ-AACGCTTCACGAATTTGCGT-3ʹ; FAM225A, forward: 5ʹ‐CCGATGGAGGTGTTGATGGATGT-3ʹ, reverse: CTGTGGCCCTTGGGCAAGTAAGT; PADI2, forward: 5ʹ- TCTCAGGCCTGGTCTCCAT -3ʹ, reverse: 5ʹ- AAGATGGGAGTCAGGGGA AT -3ʹ; and β-actin, forward: 5ʹ-GGGAAATCGTGCGTGACATTAAGG-3ʹ, reverse: 5ʹ-CAGGAAGGAAGGCTGGAAGAGTG-3ʹ.

### Western blot analysis

Total protein derived from cultured GC cells was isolated with RIPA buffer (Beyotime, China), separated by 10% SDS-PAGE, and transferred to PVDF membrane (Millipore, USA). The PVDF membrane was blocked with 5% no-fat milk in TBST at room temperature for 2 h and incubated with primary antibodies overnight (N-cadherin: CST, 1:1000; E-cadherin: CST, 1:1000; vimentin: CST, 1:1000; PADI2: CST, 1:1000; and β-actin: CST, 1:1000). The following day, the PVDF membrane was incubated with appropriate secondary antibodies at room temperature and exposed with ECL reagent (Beyotime, China) for chemiluminescent detection.

### Cell proliferation assays

For the colony formation assay, 500 cells per well were cultured in six-well plates for 2 weeks, stained with Crystal Violet, and counted. For the EdU assay, 5 × 10^3^ cells per well were cultured in 96-well plates, treated as described in the EdU assay kit (RiboBio, China) according to the protocol, stained with 4ʹ,6-diamid-ino-2-phenylindole, and visualized under a fluorescence microscope (Nikon, Japan).

### Cell migration and invasion assays

In the wound healing assay, 5 × 10^5^ cells were cultured in six-well plates and scratched with a sterile pipette tip when each well was filled with cells, and the wound size was measured at 0 and 48 h. In the transwell assay, 2 × 10^4^ cells in the upper chambers were cultured in serum-free medium, and medium with 10% FBS was added to the lower chamber. After 24 h, the migrated cells were stained with crystal violet and counted using an inverted microscope.

### Flow cytometry analysis

Treated cells were resuspended in binding buffer and stained with propidium iodide (PI) and Annexin-V-APC using the Apoptosis Detection Kit (Multisciences, China). Briefly, the harvested cells were washed with PBS, resuspended with Binding Buffer. Then, 5 μl Annexin-V-APC and 10 μl PI were added to the cell suspension and incubated for 5 min in the dark at room temperature. Data were analyzed with a flow cytometer (BD Biosciences, San Jose, CA, USA).

### Animal experiments

Treated cells were suspended in 100 μl of sterilized phosphate-buffered saline (PBS) and injected subcutaneously into the backside of female nude mice (1 × 10^6^ cells/100 μl of PBS), with five mice per group. Tumor volume was measured every 5 days and calculated as follows: volume = (length × width^2^)/2. After 25 days, the mice were euthanized, and the xenografts were weighed. All experiments were approved by the First Affiliated Hospital of NMU.

### Hematoxylin and eosin (HE) staining

Samples were embedded in paraffin, fixed in alcohol and hydrated in deionized water. The slides were then agitated with hematoxylin and washed in deionized water. Several sections were prepared, stained with hematoxylin and eosin for pathological evaluation, and visualized under a microscope (Olympus, Tokyo, Japan).

### Subcellular fractionation

A PARIS Kit (Invitrogen, USA) was used to separate RNA in the cytosolic and nuclear fractions of MKN45 or AGS cells. In brief, GC cells were incubated with lysis solution on ice for 10 min and then centrifuged for 3 min at 12,000 × *g*, cytoplasmic RNA and nuclear RNA were collected separately, then qRT-PCR was performed. U6 and β-actin were used as the nuclear and cytosolic controls, respectively.

### Fluorescence in situ hybridization (FISH)

A FISH kit (RiboBio, China) was used to perform RNA FISH as described by the manufacturer. RiboBio (Guangzhou, China) designed and synthesized the Cy3-labeled FAM225A probe. In accordance with the protocol, 1 × 10^4^ GC cells were plated into a 15 mm confocal dish. Next day, the cells were fixed, permeabilized and blocked, then blended with 20 μm probe mix to hybridize with the target sequence overnight. At the 2nd day, after treating with washing buffer to reduce the background signal, a fluorescence microscope (Olympus FV1000, Japan) was used to capture the images.

### Luciferase reporter assay

Luciferase reporter gene detection is a reporting system that uses luciferin as substrate to detect the activity of firefly luciferase. Luciferase can catalyze the oxidation of luciferin to oxyluciferin. During the oxidation of luciferin, it will emit bioluminescence. The bioluminescence released during the oxidation of luciferin can be measured by fluorescence equipment. Based on the chemiluminescence reaction between luciferase and substrate, mutant and wild-type FAM225A/PADI2 of the putative binding sites were cloned into a luciferase vector (Promega, Madison, WI, USA) and co-transfected with miR-326 mimics into GC cells. After 48 h, cells were harvested for luciferase activity analysis using the Dual-Luciferase Reporter Assay System (Promega, USA).

### Statistical analysis

SPSS 22.0 (IBM, USA) and GraphPad Prism (GraphPad Software, USA) were used to analyze the data obtained in this study. All assays were repeated independently at least three times. Experimental data are shown as mean ± standard deviation. Wilcoxon test, *χ*^2^ test, analysis of variance, Pearson correlation coefficients and two-tailed Student’s *t* test were used to estimate the significance of differences between groups. *p* < 0.05 was considered statistically significant.

## Supplementary information

table S1

figure S1

figure S2

supplementary figure legends

cddiscovery-author-contribution-form

## Data Availability

The data that support the findings of this study are available from the corresponding author upon reasonable request.

## References

[1] Sung H, Ferlay J, Siegel RL, Laversanne M, Soerjomataram I, Jemal A (2021). Global cancer statistics 2020: GLOBOCAN estimates of incidence and mortality worldwide for 36 cancers in 185 countries. CA Cancer J Clin.

[2] Chen W, Zheng R, Baade PD, Zhang S, Zeng H, Bray F (2016). Cancer statistics in China, 2015. CA Cancer J Clin.

[3] Thrumurthy SG, Chaudry MA, Chau I, Allum W (2015). Does surgery have a role in managing incurable gastric cancer?. Nat Rev Clin Oncol.

[4] Liu D, Lu M, Li J, Yang Z, Feng Q, Zhou M (2016). The patterns and timing of recurrence after curative resection for gastric cancer in China. World J Surg Oncol..

[5] Lazar DC, Avram MF, Romosan I, Cornianu M, Taban S, Goldis A (2018). Prognostic significance of tumor immune microenvironment and immunotherapy: novel insights and future perspectives in gastric cancer. World J Gastroenterol.

[6] Kapranov P, Cheng J, Dike S, Nix DA, Duttagupta R, Willingham AT (2007). RNA maps reveal new RNA classes and a possible function for pervasive transcription. Science..

[7] Mattick JS, Rinn JL (2015). Discovery and annotation of long noncoding RNAs. Nat Struct Mol Biol.

[8] Goodall GJ, Wickramasinghe VO (2021). RNA in cancer. Nat Rev Cancer.

[9] Lv Y, Wang Y, Song Y, Wang SS, Cheng KW, Zhang ZQ (2021). LncRNA PINK1-AS promotes Galphai1-driven gastric cancer tumorigenesis by sponging microRNA-200a. Oncogene..

[10] Luo Y, Zheng S, Wu Q, Wu J, Zhou R, Wang C (2021). Long noncoding RNA (lncRNA) EIF3J-DT induces chemoresistance of gastric cancer via autophagy activation. Autophagy.

[11] Monnier P, Martinet C, Pontis J, Stancheva I, Ait-Si-Ali S, Dandolo L (2013). H19 lncRNA controls gene expression of the imprinted gene network by recruiting MBD1. Proc Natl Acad Sci USA.

[12] Chen ZZ, Huang L, Wu YH, Zhai WJ, Zhu PP, Gao YF (2016). LncSox4 promotes the self-renewal of liver tumour-initiating cells through Stat3-mediated Sox4 expression. Nat Commun.

[13] Chu C, Zhang QC, da Rocha ST, Flynn RA, Bharadwaj M, Calabrese JM (2015). Systematic discovery of Xist RNA binding proteins. Cell..

[14] Statello L, Guo CJ, Chen LL, Huarte M (2021). Gene regulation by long non-coding RNAs and its biological functions. Nat Rev Mol Cell Biol.

[15] Salmena L, Poliseno L, Tay Y, Kats L, Pandolfi PP (2011). A ceRNA hypothesis: the Rosetta Stone of a hidden RNA language?. Cell..

[16] Wee LM, Flores-Jasso CF, Salomon WE, Zamore PD (2012). Argonaute divides its RNA guide into domains with distinct functions and RNA-binding properties. Cell..

[17] Kartha RV, Subramanian S (2014). Competing endogenous RNAs (ceRNAs): new entrants to the intricacies of gene regulation. Front Genet.

[18] Tay Y, Rinn J, Pandolfi PP (2014). The multilayered complexity of ceRNA crosstalk and competition. Nature..

[19] Du Z, Sun T, Hacisuleyman E, Fei T, Wang X, Brown M (2016). Integrative analyses reveal a long noncoding RNA-mediated sponge regulatory network in prostate cancer. Nat. Commun.

[20] Yilmaz M, Christofori G (2009). EMT, the cytoskeleton, and cancer cell invasion. Cancer Metastasis Rev.

[21] Yeung KT, Yang J (2017). Epithelial-mesenchymal transition in tumor metastasis. Mol Oncol.

[22] Paraskevopoulou MD, Vlachos IS, Karagkouni D, Georgakilas G, Kanellos I, Vergoulis T (2016). DIANA-LncBase v2: indexing microRNA targets on non-coding transcripts. Nucleic Acids Res.

[23] Miao YR, Liu W, Zhang Q, Guo AY (2018). lncRNASNP2: an updated database of functional SNPs and mutations in human and mouse lncRNAs. Nucleic Acids Res.

[24] Chen Y, Wang X (2020). miRDB: an online database for prediction of functional microRNA targets. Nucleic Acids Res.

[25] Agarwal V, Bell GW, Nam JW, Bartel DP (2015). Predicting effective microRNA target sites in mammalian mRNAs. Elife.

[26] Consortium EP (2012). An integrated encyclopedia of DNA elements in the human genome. Nature..

[27] Fang XY, Pan HF, Leng RX, Ye DQ (2015). Long noncoding RNAs: novel insights into gastric cancer. Cancer Lett.

[28] Zeng MS (2016). Noncoding RNAs in cancer diagnosis. Adv Exp Med Biol.

[29] Yan C, Saleh N, Yang J, Nebhan CA, Vilgelm AE, Reddy EP (2021). Novel induction of CD40 expression by tumor cells with RAS/RAF/PI3K pathway inhibition augments response to checkpoint blockade. Mol Cancer.

[30] Ho WJ, Erbe R, Danilova L, Phyo Z, Bigelow E, Stein-O’Brien G (2021). Multi-omic profiling of lung and liver tumor microenvironments of metastatic pancreatic cancer reveals site-specific immune regulatory pathways. Genome Biol.

[31] Hou Y, Zhang Q, Pang W, Hou L, Liang Y, Han X (2021). YTHDC1-mediated augmentation of miR-30d in repressing pancreatic tumorigenesis via attenuation of RUNX1-induced transcriptional activation of Warburg effect. Cell Death Differ.

[32] Chen X, Chen Z, Yu S, Nie F, Yan S, Ma P (2018). Long noncoding RNA LINC01234 functions as a competing endogenous RNA to regulate CBFB expression by sponging miR-204-5p in gastric cancer. Clin Cancer Res.

[33] Chen LL (2016). Linking long noncoding RNA localization and function. Trends Biochem Sci.

[34] Schmitt AM, Chang HY (2016). Long noncoding RNAs in cancer pathways. Cancer Cell.

[35] Huang Y, Lin Y, Song X, Wu D (2021). LINC00857 contributes to proliferation and lymphomagenesis by regulating miR-370-3p/CBX3 axis in diffuse large B-cell lymphoma. Carcinogenesis..

[36] Zhao S, Guan B, Mi Y, Shi D, Wei P, Gu Y (2021). LncRNA MIR17HG promotes colorectal cancer liver metastasis by mediating a glycolysis-associated positive feedback circuit. Oncogene.

[37] Teng F, Zhang JX, Chen Y, Shen XD, Su C, Guo YJ (2021). LncRNA NKX2-1-AS1 promotes tumor progression and angiogenesis via upregulation of SERPINE1 expression and activation of the VEGFR-2 signaling pathway in gastric cancer. Mol Oncol.

[38] Zhang X, Shi H, Yao J, Li Y, Gao B, Zhang Y (2020). FAM225A facilitates colorectal cancer progression by sponging miR-613 to regulate NOTCH3. Cancer Med.

[39] Nawaz Z, Patil V, Paul Y, Hegde AS, Arivazhagan A, Santosh V (2016). PI3 kinase pathway regulated miRNome in glioblastoma: identification of miR-326 as a tumour suppressor miRNA. Mol Cancer.

[40] Miele E, Po A, Mastronuzzi A, Carai A, Besharat ZM, Pediconi N (2021). Downregulation of miR-326 and its host gene beta-arrestin1 induces pro-survival activity of E2F1 and promotes medulloblastoma growth. Mol Oncol.

[41] Ji S, Zhang B, Kong Y, Ma F, Hua Y (2017). miR-326 inhibits gastric cancer cell growth through downregulating NOB1. Oncol Res.

[42] Bracken CP, Scott HS, Goodall GJ (2016). A network-biology perspective of microRNA function and dysfunction in cancer. Nat Rev Genet.

[43] Guo W, Zheng Y, Xu B, Ma F, Li C, Zhang X (2017). Investigating the expression, effect and tumorigenic pathway of PADI2 in tumors. Onco Targets Ther.

