# Supplementary material for: Long noncoding RNA FAM225A promotes the malignant progression of gastric cancer through the miR-326/PADI2 axis
Source: Cell Death Discov. 2022 Jan 11;8:20. doi: 10.1038/s41420-021-00809-1 (PMC8752798; doi:10.1038/s41420-021-00809-1)
Supplement: Supplementary file 1 — table S1 [file 41420_2021_809_MOESM1_ESM.docx]

| **Characteristics** | **Number** | **FAM225A expression** | | **P-value** |
| --- | --- | --- | --- | --- |
|  |  | **High** | **Low** |  |
| **Age(years)** |  |  |  |  |
| <60 | 25 | 11 | 14 | 0.451 |
| ≥60 | 43 | 23 | 20 |  |
| **Gender** |  |  |  |  |
| Male | 46 | 24 | 22 | 0.604 |
| Female | 22 | 10 | 12 |  |
| **Size** |  |  |  |  |
| <3 | 32 | 11 | 21 | **0.015*** |
| ≥3 | 36 | 23 | 13 |  |
| **Differentiation** |  |  |  |  |
| Well+Moderate | 30 | 12 | 18 | 0.143 |
| Poor+Undifferentiated | 38 | 22 | 16 |  |
| **Lymphatic metastasis** |  |  |  |  |
| Yes | 41 | 25 | 16 | **0.026*** |
| No | 27 | 9 | 18 |  |
| **Invasion depth** |  |  |  |  |
| T1+T2 | 31 | 14 | 17 | 0.465 |
| T3+T4 | 37 | 20 | 17 |  |
| **TNM stage** |  |  |  |  |
| I+II | 37 | 13 | 24 | **0.007*** |
| III+IV | 31 | 21 | 10 |  |

**Supplementary table 1**. Correlation between FAM225A expression and clinicopathological characteristics of gastric cancer patients.

*P<0.05 was considered significant
