# Supplementary figures and images for: Long noncoding RNA FAM225A promotes the malignant progression of gastric cancer through the miR-326/PADI2 axis
Source: Cell Death Discov. 2022 Jan 11;8:20. doi: 10.1038/s41420-021-00809-1 (PMC8752798; doi:10.1038/s41420-021-00809-1)

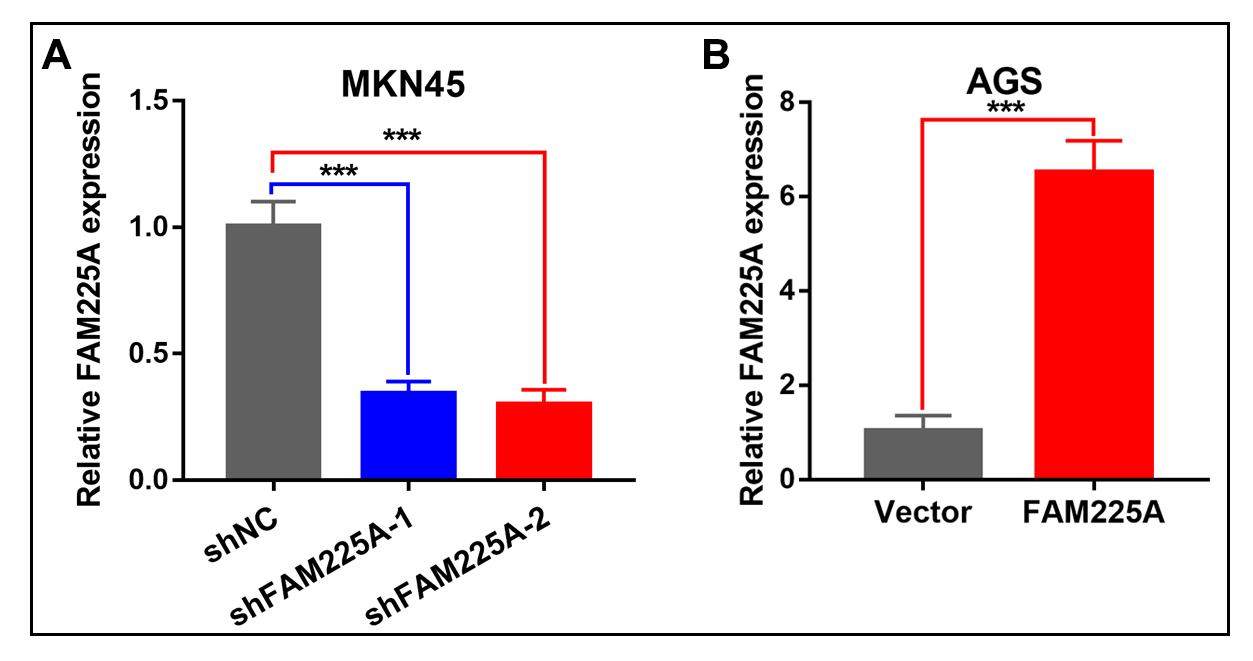

Supplement: Supplementary file 2 — figure S1 [file 41420_2021_809_MOESM2_ESM.tif]

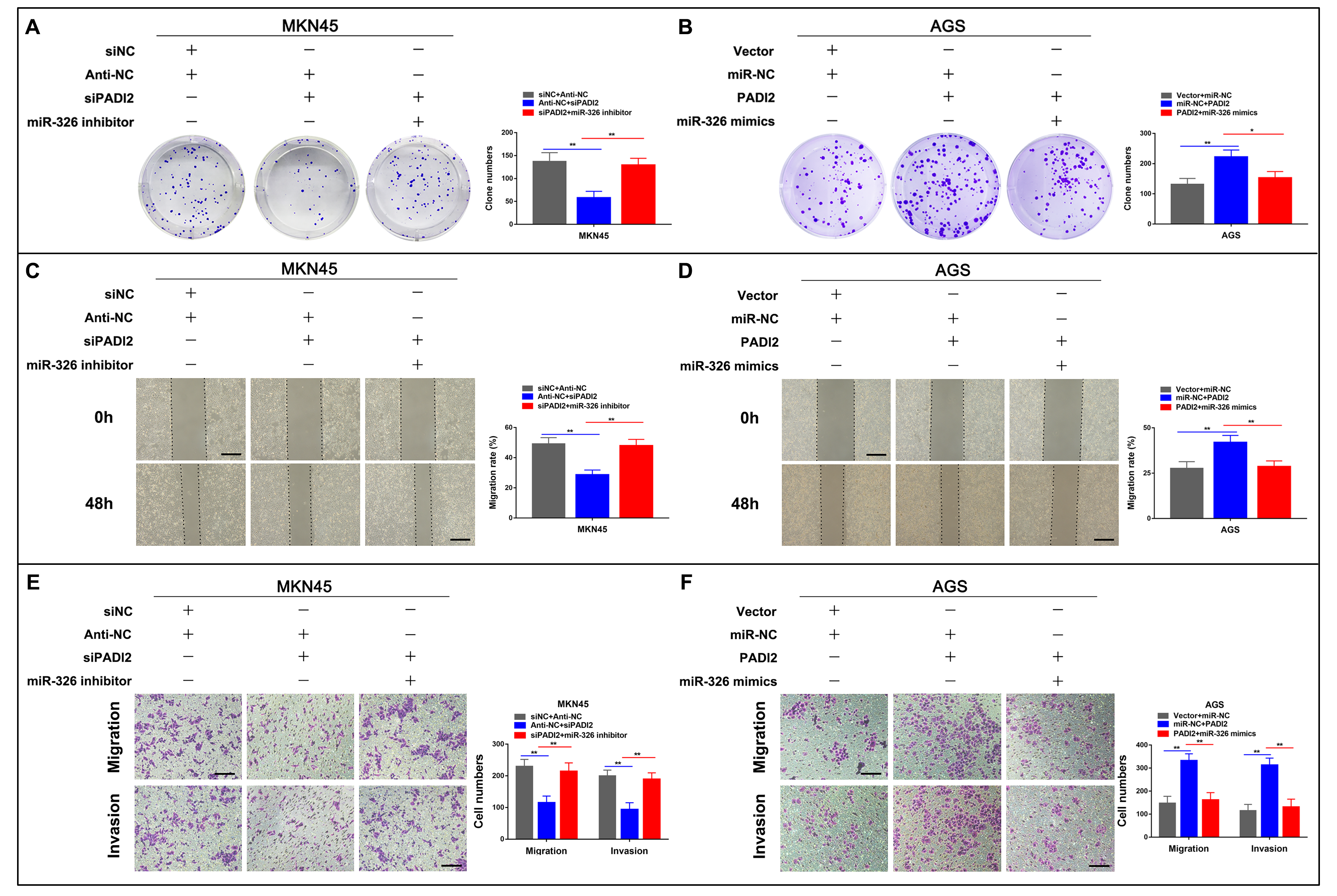

Supplement: Supplementary file 3 — figure S2 [file 41420_2021_809_MOESM3_ESM.tif]
