# Supplementary material for: Long noncoding RNA FAM225A promotes the malignant progression of gastric cancer through the miR-326/PADI2 axis
Source: Cell Death Discov. 2022 Jan 11;8:20. doi: 10.1038/s41420-021-00809-1 (PMC8752798; doi:10.1038/s41420-021-00809-1)
Supplement: Supplementary file 4 — supplementary figure legends [file 41420_2021_809_MOESM4_ESM.docx]

**Long noncoding RNA FAM225A promotes the malignant progression of gastric cancer through the miR-326/PADI2 axis**

Xiang Ma^1^, Gang Wang^1^, Hao Fan^1^, Zengliang Li^1^, Wangwang Chen^1^, Jian Xiao^1^, Peidong Ni^1^, Kanghui Liu^1^, Kuan Shen^1^, Yuanhang Wang^1^, Zekuan Xu^1^, Li Yang^1,2^

^1^ Department of General Surgery, the First Affiliated Hospital of Nanjing Medical University, Nanjing, Jiangsu Province, China

^2^ Department of General Surgery, Liyang People’s Hospital, Liyang Branch Hospital of Jiangsu Province Hospital, Liyang, Jiangsu Province, China

**Supplementary Figure Legends**

Supplementary Figure 1.

A. qRT-PCR analysis of FAM225A mRNA in MAKN45 cells treated with si-FAM225A. B. Relative expression of FAM225A confirmed by qRT-PCR in AGS cells with FAM225A overexpression. P-values were obtained by Student’s t test. Data are presented as means ± SD. ***p < 0.001.

Supplementary Figure 2.

A,B. Colony formation assays was used to detect the cell proliferation ability after transfecting MAN45 cells with negative control, siPADI2 or siPADI2 + miR-326 inhibitor, and transfecting AGS cells with negative control, PADI2 or PADI2 + miR-326 mimics. C-F. The wound healing and transwell assays were used to detect the cell migration and invasion ability after transfecting GC cells (scale bar: 200 μm for transwell assay, 100 μm for wound healing assay). P-values were obtained by Student’s t test. Data are presented as means ± SD. *p < 0.05, **p < 0.01.
